# 

**Published:** 1912-02-03

**Authors:** 


### Appointments.

The Election Committee of the General Hospital, Birmingham, held on Friday last week, Sir John C. Holder, Bart. (Vice-President), in the chair, elected Dr. James William Russell, M.A., M.D., B.C. Cantab., F.R.C.P. Lond., Honorary Physician for a term of fifteen years. Dr. Russell, the son of the late Dr. James Russell, who was honorary physician of the hospital from January 1859 to January 1885, was appointed resident medical officer of the General Hospital in March 1891, and in March 1892, was appointed assistant physician, so that he has already served the hospital for nearly twenty-one years. Dr. Russell is also consulting physician to the Birmingham Midland Ear and Throat Hospital, the Royal Institution for the Blind, the Midland Counties Institution, and the Sutton Coldfield Cottage Hospital. He is also assistant to the Chair of Medicine in the University of Birmingham.

The following appointments at the Manchester Royal Infirmary have been confirmed; Mr Frederick H. Westmacott, F.R.C.S., to be Assistant Surgical Officer to the Aural Department; Mr. Newton Matthews, M.B., Ch.B. Vict., to be House Physician; and Mr. C. Cathcart, M.B., Ch.B. Edin., to be Assistant Medical Officer at Barnes Convalescent Home, Cheadle. The following re-appointments have also been made: Mr. D. E. Core, M.D. Vict., M.R.C.P., as Resident Medical Officer; Mr. F. E. Tylecote, M.D., M.R.C.P., as Medical Registrar; and Mr. E. E. Hughes, M.B., Ch.B. Vict., as Resident Medical Officer at Barnes Convalescent Home.

### OUR BUREAU OF INFORMATION.

### Our List of Approved Homes.

### Rules for Correspondents.

Everyone who uses or wishes to help our Bureau of Information must be kind enough to observe the following rules:—

(1) Postal replies cannot be given. Exception will only be made in very special cases at the Editor's discretion.

(2) Queries or answers relating to different cases must be written, or preferably typed, on separate sheets of paper, and the writing must never be crossed.

(3) Questions and replies received not later than Wednesday will, as far as possible, be dealt with in our issue of the following Saturday week.

(4) Letters must have attached the name and address of the sender, with a pseudonym for publication if preferred.

(5) Applications referring to non-dependents where the need relates to business or employment must be accompanied by a postal order for 5s., when the requirement shall have publicity in these columns.

(6) All applicants for insertion in our list of Approved Homes must send a registration fee of five shillings. The omission of this leads to delay and gives avoidable trouble.

(7) *All communications* for this department *must be accompanied by the coupon* to be found at the bottom of the third page of the cover on the inside, and should be addressed to the Editor of the Bureau of Information, c/o The Hospital, 29 Southampton Street, Strand, London, W.C.

### Florence Nightingale Memorial.

We have to acknowledge the receipt of the following contributions :—Officers and Nursing Staff of the Albert Dock Branch of the Seamen's Hospital Society, £3 1s.

Received by Mr. G. Q. Roberts : Royal Army Medical Corps Fund, £21; Mrs. Earle's Collection (additional), £5; Mrs. J. Maude, £2; Anon., £2; Collection at General Hospital, Birmingham, £1 10s.; Holroyd Chaplin, Esq., £1 1s.; Mrs. C. Ursinus, £1 1s.; Miss Benecke, £1 1s.; E. H., £1 1s.; Miss Ainger, 10s.; Collected in Mr. Linder's Shipwrights' Gang, H.M. Dockyard, Chatham, 2s. 7d.

### The Week's Novelties.

### A NEW INVALID LIFTER.

Appliances for lifting invalids or maintaining them in various positions have come and gone, as every hospital secretary knows. But on the whole it is true to say that the later the design the more suitable it is. Mr. Arthur Skeffington's appliances have been often described to our readers, and his latest deserves a note. It is known as "Skeffington's Inclinator," and as its peculiar device requires examination, all curious to see his improvement should write for particulars at 49 Ulundi Road, S.E., mentioning The Hospital, which this week publishes an announcement and illustration which will be found on reference to our advertisement columns. The "Skeffington Sacrum Lifter" and its B pattern for adjusting to a private bedstead are especially worth notice. It is only fair to add further lifters for special purposes will shortly be brought out. We do not propose to anticipate in detail, but it may be said that one of them is likely to appeal to workhouse infirmaries, for which the solution of a particular problem in reference to elderly patients has been attempted.

### NATURAL SODA-WATER.

(Cutlack and Co., Peterborough.)

This is a pure natural soda-water. Its alkalinity is pronounced; it is not merely water saturated with $CO_2$ gas derived from bicarbonate of soda. It contains the bicarbonate itself in appreciable quantity (in the sample tested by us this amounted to approximately 70 grains per gallon). In addition it contains small quantities of carbonate of lime and magnesia, and holds in solution a fair amount of sulphate and chloride of soda, though these salts are not present in such quantity as to give the water a markedly saline or bitter taste. On the contrary, they enhance the biting sharpness which is communicated by the gas that the water contains. The sample examined by us was quite pure; it contained no trace of deleterious matter or of organic ammonia. It blended well with both whisky and brandy, slightly less well with burgundy, but equally well with lime-juice. As a table drink we consider this water one of the very best obtainable. Its taste is decidedly above the average, and its purity and sparkle should make it popular with everyone who has tried it.

